# Calpain Activity and Toll-Like Receptor 4 Expression in Platelet Regulate Haemostatic Situation in Patients Undergoing Cardiac Surgery and Coagulation in Mice

**DOI:** 10.1155/2014/484510

**Published:** 2014-08-31

**Authors:** Jui-Chi Tsai, Yi-Wen Lin, Chun-Yao Huang, Feng-Yen Lin, Chien-Sung Tsai

**Affiliations:** ^1^Graduate Institute of Medical Sciences, National Defense Medical Center, Taipei 114, Taiwan; ^2^Department of Internal Medicine, School of Medicine, College of Medicine, Taipei Medical University, Taipei 110, Taiwan; ^3^Division of Cardiology, Department of Internal Medicine, Taipei Medical University Hospital, Taipei 110, Taiwan; ^4^Institute of Oral Biology, National Yang-Ming University, Taipei 112, Taiwan; ^5^Division of Cardiovascular Surgery, Tri-Service General Hospital, National Defense Medical Center, Taipei 114, Taiwan

## Abstract

Human platelets express Toll-like receptors (TLR) 4. However, the mechanism by which TLR4 directly affects platelet aggregation and blood coagulation remains to be explored. Therefore, in this study, we evaluated the platelet TLR4 expression in patients who underwent CABG surgery; we explored the correlation between platelet TLR4 expression and the early outcomes in hospital of patients. Additionally, C57BL/6 and C57BL/6-*Tlr* ^*LPS*−/−^ mice were used to explore the roles of platelet TLR4 in coagulation by platelet aggregometry and rotation thromboelastometry. In conclusion, our results highlight the important roles of TLR4 in blood coagulation and platelet function. Of clinical relevance, we also explored novel roles for platelet TLR4 that are associated with early outcomes in cardiac surgery.

## 1. Introduction

Toll-like receptors (TLRs) are type I transmembrane receptors that are expressed on the cell membrane and are critical for the induction of downstream signals during innate immune responses to bacterial components [1]. TLR4 is expressed in many cells, including endothelial cells, keratinocytes, epithelial cells, macrophages, neutrophils, and dendritic cells [2–4]. Previous evidences demonstrated that platelet TLR4 expression modulates lipopolysaccharide- (LPS-) stimulated platelet aggregation [5] and tumor necrosis factor-alpha (TNF-*α*) production [6]. In addition, circulating platelet counts decrease precipitously during sepsis and the degree of thrombocytopenia associates with the serious consequence of sepsis [7]. Furthermore, platelet counts decreased under sepsis because of well-established migration into the liver and lungs [7–9]. Andonegui et al. demonstrated that platelet TLR4 is essential for platelet migration into the lungs in mice with LPS-induced sepsis [10]. LPS accelerates collagen- or thrombin-induced aggregation in platelets in* in vitro* study; this acceleration is associated with expression of TLR4 [5]. It has been reported that the function of platelet TLR4 is to activate neutrophil extracellular traps in septic blood [11], and histones may promote the generation of thrombin via platelet TLR4 [12]. Thus, scientists proposed that platelet TLR4 has important roles in platelet migration, adhesion, destruction, and attraction. Although the evidence indicates that TLR4 not only regulates platelet migration and shape change but also indirectly affects their aggregation, it remains to be explored whether TLR4 expression directly affects platelet aggregation and blood coagulation.

Previous studies demonstrated that the level of TLR4 expression on cells is associated with the early outcome of patients undergoing major surgery. In 2002, Dybdahl et al. demonstrated that open heart surgery induces an inflammatory response and the release of heat-shock protein 70 via TLR4 signaling [13]. Recently, our group demonstrated that the level of TLR4 expression on monocytes is associated with early outcomes in coronary artery bypass graft (CABG) surgery patients [9]. Previous evidence indicated that platelets possess a biological function with clinical correlation [14]. It remains unclear whether the platelets regulate both blood coagulation and systemic inflammation in surgery and the expression of TLR4 on platelets is affected by the type of cardiac surgery, which may be associated with a patient's early outcome in hospital. CABG with conventional cardiopulmonary bypass (CPB) is widely used in cardiovascular surgery. Although stopping the heart and temporarily replacing its function with the heart-lung machine has associated risks, conventional CPB permits surgeons to perform coronary revascularization on a still, bloodless heart. However, evidence suggests that platelet count, aggregation, and shape change significantly decreased after conventional CPB [15]. Platelet activation and platelet injury are well-recognized phenomena occurring after cardiac surgery with CPB that may contribute to pathological postoperative bleeding [16] and systemic oxidative stress/inflammation. To control the systemic oxidative stress and inflammatory responses as well as decrease the occurrence of postoperative complications, cardiac surgeons have devoted their efforts to developing various surgical processes, such as the off-pump technique or beating-heart CPB technique for coronary surgery. Although the previous literatures indicated that off-pump technique has become an established and feasible procedure and offers a great benefit in CABG patients [12, 17–21], the other studies also hold contrary opinion [22, 23]. Regardless of the technique of coronary surgery, clarifying the actual molecular mechanisms of impact of the platelet function is the only way to promote the patients' outcomes.

It is well known that calpain plays important roles in the physiological activation of platelets. Calpain activation regulates SNAREs (proteins involved in the degranulation of platelets) and SERCA-2 (an intracellular pump required for Ca^2+^ signaling in platelets). Previous evidences demonstrated that cardiac surgery induces oxidative stress, and oxidative stress may inhibit calpain activity [24]. Based on our evidence, the calpain-myosin 9-Rab7b axis is responsible for the regulation of TLR4 in activated platelets, which is involved in platelets function [25]. Therefore, we evaluated the platelet TLR4 expression in patients who underwent CABG surgery with conventional CPB or off-pump technique and analyzed the correlation between platelet calpain activation, TLR4 expression, and drainage loss in hospital. Additionally, in order to confirm the critical role of TLR4 on platelets function, we analyzed the coagulation in C57BL/6 and C57BL/6-*Tlr* ^*LPS−/−*^ mice.

## 2. Materials and Methods

### 2.1. Clinical Observations

#### 2.1.1. Ethics and Patient Inclusion

This study was approved by the ethics committee at Tri-Service General Hospital. Written informed consent was obtained from 23 patients undergoing elective CABG surgery. The patients were assigned to the off-pump technique and conventional cardiopulmonary bypass (CPB) groups. CPB and off-pump technique was used in 17 and 6 patients, respectively. Patients would not be included if they experienced a decreased cardiac ejection fraction (low than 50%), had undergone cardiac surgery, had a history of cardiogenic shock, had used extracorporeal membrane oxygenation (ECMO), received an intra-aortic balloon pump (IABP), or placed on a ventilator before surgery. Additionally, patients with chronic bronchitis, autoimmune diseases, cancer, asthma, or rheumatoid arthritis or who were receiving anti-inflammatory drugs administration were excluded.

#### 2.1.2. Anesthesia, Heparinization, Conventional CPB, and Off-Pump Technique

All the techniques were described in the previous report [26]. Patients received anesthesia, induced with thiopental, and maintained with isoflurane in oxygen. Heparin was administered before applying the stabilization device or before cannulation. All patients underwent a median sternotomy during surgery. The blood pressure was monitored using an arterial catheter. Upon discontinuation of the CPB circuit or completion of proximal anastomoses, protamine sulfate was used to neutralize the heparin. No antifibrinolytic drugs were used in these patients.

#### 2.1.3. Blood Sample Collection and Biolaboratory Studies

Blood was collected from the arterial catheter. All samples were collected in 3.8% sodium citrate-containing tubes. The blood samples were collected at two time points: (1) preincision and (2) at the end of the operation. The leukocytes and platelet counts were measured, and blood biolaboratory studies were performed.

#### 2.1.4. Flow Cytometry Analysis

Whole blood samples were collected from all patients at the indicated time points and analyzed immediately. Fluorescein isothiocyanate- (FITC-) conjugated mouse anti-human CD41a antibodies (Becton Dickinson, San Jose, CA, USA) and phycoerythrin- (PE-) conjugated monoclonal mouse anti-human TLR4 antibodies (clone: HTA125; Biolegend, San Diego, CA, USA) were added to 50 *μ*L whole blood and incubated in the dark for 20 min. The CD41a-positive cells were separated and detected using a BD FACSCanto II flow cytometer (BD Biosciences, Mountain View, CA, USA) with BD FACSDiva software (Becton Dickinson Immunocytometry Systems, San Jose, CA, USA). A total of 3 × 10^4^ CD41a-positive cells were gated for the analysis of membrane TLR4 expression.

#### 2.1.5. Preparation of Washed Human Platelets and Western Blot Analysis

Washed platelets were isolated according to previous report [27]. The pelleted platelets were lysed using cell lysis buffer. Western blot analysis was performed using 50 *μ*g samples of protein to evaluate calpain-1 expression. The cell lysates were subjected to 7% SDS-polyacrylamide gel electrophoresis. The protein was electrophoretically transferred to polyvinylidene difluoride membrane and then blocked with 5% milk in Tris-buffered saline solution (20 mM Tris-HCl pH 7.5, 138 mM NaCl, and 0.2% NP-40) containing 0.2% Tween-20. The membranes were probed using monoclonal rabbit anti-human calpain antibody (clone: HPR3319; Epitomics Co., Burlingame, CA, USA), incubated with horseradish peroxidase-conjugated anti-rabbit IgG (Amersham, Arlington Heights, IL, USA), and developed using the enzyme-linked chemiluminescence detection reagents (Millipore, Bedford, Mass., USA). *β*-actin was used as a loading control; the monoclonal mouse anti-human *β*-actin antibody (clone: ACTN05/C4) was obtained from GeneTex Co. (Irvine, CA, USA).

### 2.2. Animal Study

#### 2.2.1. C57BL/B6 and C57BL/6-*Tlr* ^*LPS−/−*^ Mice

Six male C57BL/B6 mice were purchased from BioLASCO Co., Ltd., (Yi-Lan, Taiwan). Six male C57BL/6-*Tlr* ^*LPS−/−*^ mice (a TLR4-knockout mouse homozygous for the defective LPS-response deletion allele* Tlr4* ^*lps-del*^) were purchased from the Jackson Laboratory (JAX, 003752, Bar Harbor, ME, USA). This study was carried out in strict accordance with the recommendations in the Guide for the Care and Use of Laboratory Animals of the National Institutes of Health (NIH Publication number 85-23, revised 1996). The protocol was approved by the Committee on the Ethics of Animal Experiments of the Taipei Medical University (Permit Number: LAC-100-0056). All mice were kept in microisolator cages on a 12-h day/night cycle and fed a commercial mouse chow diet (Scientific Diet Services, Essex, UK) with water* ad libitum*. The blood samples for biochemical measurements were collected without sedation from the mandibular artery in sodium citrate-containing tubes and separated by centrifugation.

#### 2.2.2. Platelet Aggregometry

Whole blood was collected from the mice through cheek-pouch bleeds and anticoagulated with 3.8% sodium citrate. Platelet-rich plasma (PRP) was obtained from the collected blood by centrifugation at 280 ×g for 8 min. The PRP was gently transferred to a fresh tube, and the centrifugation was repeated at 280 ×g for 4 min. The platelets were isolated by filtering the resulting PRP through a Sepharose 2B column (Sigma-Aldrich, St. Louis, MO, USA) equilibrated with Ca^2+^-free Tyrode's buffer. The washed platelets were counted using an automated blood cell counter KX-21N (Sysmex, Kobe, Japan). Platelet aggregation was performed using the turbidimetric method with a Chrono-Log Model 560-Ca Dual sample Lumi-Ionize calcium aggregometer (Chrono-Log, Havertown, PA, USA). Approximately 500 *μ*L of a washed platelet solution containing 3 × 10^5^ platelets/*μ*L in Ca^2+^-free Tyrode's buffer was added to a silicon-treated glass cuvette for each experiment. The platelets were kept warm at 37°C and stirred at 1200 rpm. Platelet aggregation was measured for 5 minutes following the addition of 0.1 U/mL thrombin. The percent aggregation was calculated using AGGRO/LINK software (Chrono-Log, Havertown, PA, USA). All buffers were prewarmed before use and platelets were kept warm at 37°C all along the study.

#### 2.2.3. Rotation Thromboelastometry (ROTEM Analyses)

In accordance with previous studies, two ROTEM analyses were performed within minutes after the sample was collected, as indicated by the manufacturer's instructions (Pentapharm, Munich, Germany). We performed both an extrinsically activated assay using recombinant tissue factor (EXTEM) and an extrinsically activated test using recombinant tissue factor with cytochalasin D added (FIBTEM). The following parameters were measured using ROTEM: clotting time (CT, the time from the start of the assay to clot formation with an amplitude of 2 mm), clot formation time (CFT, the time from the end of CT (amplitude of 2 mm) to a clot firmness of 20 mm), maximum clot firmness (MCF, the peak strength of the clot), and maximum clot elasticity (MCE, which is commonly used to assess clot strength). The platelet component of clot strength was calculated using the following equation: MCF_platelet_ = MCF_EXTEM_ − MCF_FIBTEM_, MCE_platelet_ = MCE_EXTEM_ − MCE_FIBTEM_, and MCE = (MCF × 100)/100 − MCF [28].

### 2.3. Statistical Analysis

The data are expressed as the mean ± SD. Statistical comparisons between groups were computed using Student's *t*-tests and one-way ANOVA followed by the Dunnett's test. For all statistical evaluations, differences in data with *P* values of <0.05 were considered statistically significant. All analyses were performed using the SPSS 16 statistical package (SPSS Inc., Chicago, IL, USA).

## 3. Results

### 3.1. Preoperative and Perioperative Characteristics in Patients Who Underwent Elective CABG

The clinical characteristics of the patients before surgery are listed in Table 1. The preoperative characteristics of the two groups were similar, including in age, body weight, height, hypertension, hypercholesterolemia, and peripheral vascular disease. The percentages of EF in the conventional CPB and off-pump technique groups were 59.7 ± 9.2% and 63.5 ± 8.3%, respectively. None of the patients in the off-pump group had diabetes mellitus or peripheral vascular disease. In addition, no patient who underwent elective CABG with conventional CPB had previously experienced stroke or myocardial infarction.

The perioperative characteristics are shown in Table 2. For patients in the CPB group, the mean total CPB time was 119.1 ± 12.4 min, the aortic clamping (ischemia) time was 65.6 ± 5.2 min, and the minimal esophageal temperature was maintained at 26.1 ± 0.4°C. Additionally, the patients in the conventional CPB group received 30476.2 ± 1708.0 units of heparin. The patients who underwent the off-pump technique maintained a minimal esophageal temperature of 35.8 ± 0.3°C and were administered 28142.9 ± 4532.6 units of heparin. There were no significant differences in the number of grafts or amount of heparin used between the two groups.

### 3.2. Biochemical Analyses in Patients Who Underwent Elective CABG

Table 3 showed the biochemical data for patients. The percentages of circulating lymphocytes, monocytes, basophils, and neutrophils were not significantly different between the conventional CPB and off-pump technique groups before surgery. The percentages of neutrophil numbers increased significantly in both the conventional CPB group (from a basal level of 60.9 ± 9.2% to 85.9 ± 3.6%) and the off-pump technique group (from a basal level of 64.7 ± 8.0% to 86.2 ± 4.3%). The number of lymphocytes decreased significantly after coronary surgery with conventional CPB (from a basal level of 28.3 ± 9.2% to 7.5 ± 3.2%, resp.) and with the off-pump technique (from a basal level of 26.1 ± 7.7% to 6.8 ± 2.7%, resp.). The eosinophils, basophils, and monocytes did not significantly differ between the two groups after surgery. Additionally, the number of platelets was slightly reduced in the conventional CPB group. Biochemical analyses indicated that liver function (levels of glutamate oxaloacetate transaminase, AST, and glutamic pyruvic transaminase, ALT), kidney function (levels of blood urea nitrogen, BUN, and creatinine), and inflammation (level of C-reactive protein, CRP) indices did not deteriorate after cardiac surgery. Patients who underwent conventional CPB exhibited higher levels of troponin I after surgery, compared with the off-pump technique group. However, the mean value of creatine kinase-MB isoform (CK-MB) was not significantly different between the conventional CPB and off-pump technique groups.

### 3.3. Patients in the Off-Pump Technique Group Exhibited Higher TLR4 Expression and Calpain Activity Than Patients in the Conventional CPB Group

**Figure 1:**
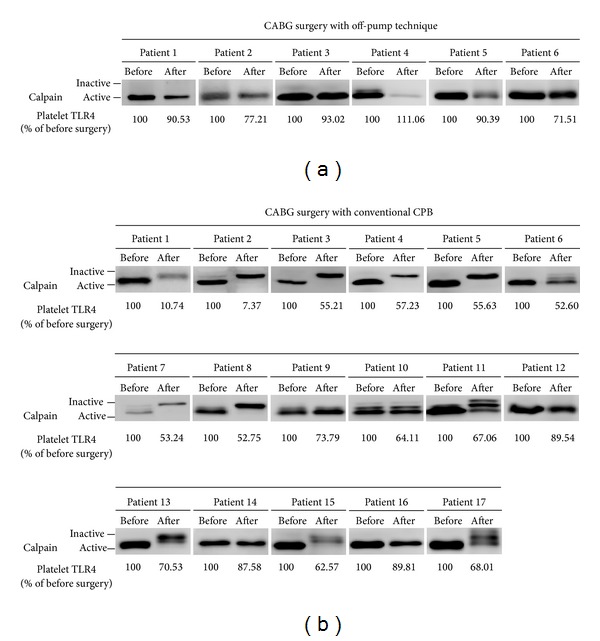
CABG surgery with conventional CPB decreases calpain activity in platelets. (a) Total protein was isolated from washed platelets collected from patients who underwent the off-pump technique. The western blot results demonstrate the calpain activity before and after CABG surgery. (b) Total protein isolated from washed platelets collected from patients who underwent conventional CPB. The western blot results demonstrate the calpain activity before and after CABG surgery.

Washed platelets were isolated from all patients. Flow cytometry was performed to analyze the TLR4 expression, and western blot analysis was used to analyze the calpain activity.  Table 4 indicates that patients in the conventional CPB group possessed lower TLR4 expression (CPB: 68.48 ± 5.64% of preincision versus off-pump technique: 89.0 ± 4.8% of preincision, *P* < 0.05), less drainage loss (CPB: 68.5 ± 5.6% of preincision versus off-pump technique: 89.0 ± 4.8% of preincision, *P* < 0.001), and higher pRBC transfusion volume (CPB: 5.5 ± 0.5 units versus off-pump technique: 1.1 ± 1.1 units, *P* = 0.001) compared with the off-pump technique group. The platelet transfusion volume, duration of ICU stay, and duration of fever were not significantly different between the conventional CPB and off-pump technique groups after CABG surgery. According to our recent results [25], TLR4 expression on platelets is mediated by the activity of calpain.  Figure 1 shows the calpain activity in all patients before and after the CABG surgery was performed. In general, inactivated calpain appears as a 80 kDa of single band; in contrast, calpain has a higher mobility 78 kDa band resulted from autolysis under activated situation, which is believed to be the more active form of calpain. Before CABG surgery, all patients in both the off-pump technique and conventional CPB groups expressed active calpain. While undergoing CABG surgery, all patients in the off-pump technique group continued to express active calpain. In contrast, 76.47% of patients (patient numbers 1, 2, 3, 4, 5, 6, 7, 8, 10, 11, 13, 15, and 17) in the conventional CPB group expressed inactive calpain after CABG surgery. Additionally, the ratio of platelets TLR4 expression after surgery was demonstrated in Figure 1. Combined with the data of TR4 expression and calpain expression (Figure 1), the information shown in Table 5 indicates that patients in the conventional CPB group with active calpain possessed higher levels of TLR4 on platelets, less drainage loss, lower platelet transfusion volumes, shorter ICU stay durations, and shorter fever duration after CABG surgery (G1 versus G2, *P* < 0.05 were considered statistically significant). Although the calpain remained in an active state, the amounts of platelet transfusion were reduced in the off-pump technique group compared with the conventional CPB group (G1 versus G3, *P* < 0.05, considered statistically significant). Interestingly, compared with patients who underwent the off-pump technique, the patients possessed a lower level of TLR4, higher volumes of drainage loss, and pRBC/platelet transfusion as well as longer ICU stay duration and shorter fever duration in the conventional CPB group with inactive calpain (G2 versus G3, *P* < 0.05, considered statistically significant). These results indicate that in CABG surgery with either off-pump technique or conventional CPB, patients with higher level of TLR4 may experience better early outcomes (duration of ICU stay and fever, volume of drainage loss, and pRBC/platelet transfusion) in hospital than the patients with lower level of TLR4, which may be mediated by activation of calpain on platelets.

### 3.4. TLR4 Is Associated with Platelet Function in Mice

A previous study demonstrated that platelet TLR4 modulates coagulation by recognizing extracellular histones that promote thrombin generation [12].

**Figure 2:**
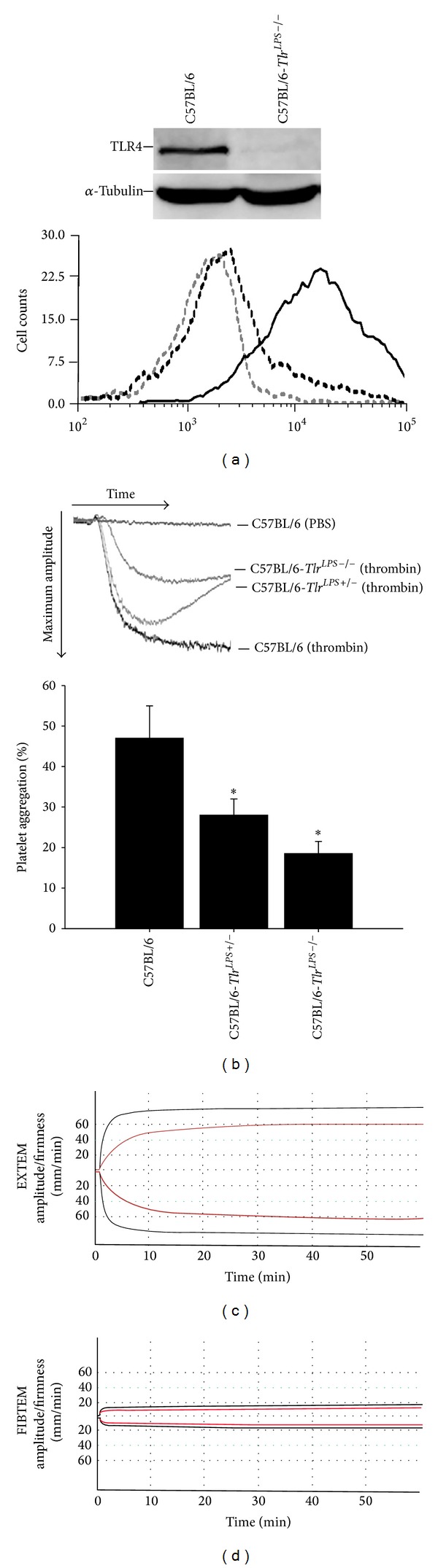
Platelet TLR4 is associated with platelet function in C57BL/6 mice. (a) TLR4 protein expression on the surface of platelets in C57BL/6 and C57BL/6-*Tlr* ^*LPS*−/−^ mice was analyzed by western blot analysis (upper graph) and flow cytometry (button graph). Negative control analyses were performed in the absence of specific PE-conjugated anti-TLR4 antibody (gray outline-hollow graph). C57BL/6 mice (black graph), but not C57BL/6-*Tlr* ^*LPS*−/−^ mice (black outline-hollow graph), express TLR4 protein in platelets. (b) Platelet aggregation was analyzed using aggregometry. The upper graph shows C57BL/6, C57BL/6-*Tlr* ^*LPS*−/−^, and C57BL/6-*Tlr* ^*LPS*−/−^ mouse platelets with 0.1 U/mL thrombin or PBS treatment. The data at the bottom illustrate the mean ± SD (*n* = 6). *P < 0.05 indicates a significant difference compared with the C57BL/6 group. (c and d) ROTEM was performed. The thromboelastometry graph shows the curves of EXTEM (c) and FIBTEM (d) in C57BL/6 (black line) and C57BL/6-*Tlr* ^*LPS*−/−^ (red line) mice.

Additionally, Tables 1–5 indicate that calpain and TLR4 expression in platelets are associated with patients' drainage loss in cardiac surgery. Therefore, we further explored whether the TLR4 on platelets is associated with coagulation in mice. The wild-type C57BL/6, C57BL/6-*Tlr* ^*LPS*+/−^, and C57BL/6-*Tlr* ^*LPS*−/−^ mice were used in this study. The endogenous level of TLR4 expression in the mouse platelets was first measured using western blot analysis and flow cytometry (Figure 2(a)). The C57BL/6-*Tlr* ^*LPS*−/−^ mice did not express TLR4 on their platelets. Incubating wild-type C57BL/6 mouse platelets with 0.1 U/mL thrombin resulted in approximately 47.4 ± 5.4% aggregation within 10 minutes, whereas treating platelets with PBS did not induce aggregation (Figure 2(b)). Interestingly, platelets from C57BL/6-*Tlr* ^*LPS*+/−^ or C57BL/6-*Tlr* ^*LPS*−/−^ mice had reduced aggregation abilities after thrombin stimulation compared with those from wild-type mice (28.1 ± 3.7% for C57BL/6-*Tlr* ^*LPS*+/−^ and 18.5 ± 2.5% for C57BL/6-*Tlr* ^*LPS*−/−^). In recent years, ROTEM has been used to provide a comprehensive overview of the entire clotting process by measuring the coagulation function of platelets. Figures 2(c) and 2(d) show the hemostatic curve from the ROTEM analysis. An extrinsically activated assay using recombinant tissue factor (EXTEM) indicated no significant differences in the coagulation time or clot formation time between C57BL/6, C57BL/6-*Tlr* ^*LPS*+/−^, and C57BL/6-*Tlr* ^*LPS*−/−^ mice. The amplitude at 5 to 30 minutes and M*CF* were significantly lower in* C57BL/6-Tlr* ^*LPS*+/−^ compared with* C57BL/6* (Table 6). An extrinsically activated test using recombinant tissue factor plus cytochalasin D (FIBTEM) demonstrated that C57BL/6-*Tlr* ^*LPS*−/−^ mice have a lower coagulation time, lower amplitude at 15 to 30 minutes, and lower MCF than C57BL/6 mice (Table 7). According to a previous report [28], MCF and MCE are commonly used to assess the platelet component of clot strength.  Table 8 demonstrates that both C57BL/6-*Tlr* ^*LPS*+/−^ and C57BL/6-*Tlr* ^*LPS*−/−^ mice have significantly lower MCFs (48.80 ± 3.27 mm and 48.50 ± 3.00 mm, resp.) and MCEs (190.89 ± 20.79 mm and 218.37 ± 19.54 mm, resp.) than C57BL/6 mice (MCF: 58.80 ± 4.67 mm; MCE: 290.85 ± 10.99 mm). These results suggest that platelet TLR4 is associated with aggregation and clot strength in C57BL/6 mice.

## 4. Discussion

TLR4 plays important role in platelet migration, adhesion, destruction, and attraction. In 2005, Andonegui et al. first determined that platelets express TLR4 on their surfaces and that this phenomenon is associated with the occurrence of thrombocytopenia [10], indicating that the TLR4-mediated signaling pathway is involved in the cellular function of platelets. Additionally, platelets may respond to endotoxin and ensnare bacteria through TLR4 to activate neutrophil extracellular traps [11, 29], promote migration into the lung or liver [8, 10], and decrease the production of RANTES, angiogenin, and PDGF-AB from platelets in severe septic blood [30]. Although previous investigators demonstrated that platelets regulate inflammation via TLR4 surface expression, few studies investigated the relationship between platelet TLR4 and coagulation. Explored ADP-induced heat shock protein (HSP) 27 secretion and phosphorylation from granules in platelets [31], which mediates platelet aggregation [32]. Moreover, the effects of HSP27 in cells were mediated through the TLR4 pathway [33]. Platelet TLR4 modulates coagulation by recognizing extracellular histones that promote thrombin generation [12].

Interestingly, LPS did not increase human platelet aggregation* in vitro*; nevertheless, LPS accelerates the collagen/thrombin-stimulated aggregation of platelets [5]. This mechanism was mediated by TLR4 expression on platelets, and the effect of LPS on platelets was indirect. In addition to TLR4, platelets also express downstream components of the TLR4-related signaling pathway complex, including TLR4/MD2 and myeloid differentiation primary response gene (MyD)88 [5]. Through the production of interleukin- (IL-) 6, prostaglandin E2, and tumor necrosis factor (TNF)-*α*, LPS induces platelet aggregation mediated by TLR4/MD2/MyD88 complex formation, mitogen-activated protein kinases (MAPKs), nuclear factor-kappa* B* (NF-*κ*B) activation, and cGMP production [5, 7, 34]. Although many reports speculated that TLR4 affects platelet aggregation and regulates platelet function, we are the first group to indicate that the calpain-myosin 9-Rab7b axis is liable to the regulation of TLR4-containing α-granule trafficking in thrombin-induced platelets [25]. Furthermore, we demonstrated the differences of platelet function in C57BL/6, C57BL/6-*Tlr* ^*LPS*+/−^, and C57BL/6-*Tlr* ^*LPS*+/−^ mice in this text, indicating the critical roles of TLR4 in platelets. We speculate that the probable mechanisms of the impact of TLR4 on clot strength in animal experiments may be mediated by HSP27 or others, and we are currently investigating the cellular and molecular mechanisms involved in this phenomena.

In clinic, patients who underwent CABG surgery with inactive calpain and lower platelet TLR4 expression experienced worse early outcomes in hospital than those with active calpain and higher platelet TLR4 expression. These results indicate that the levels of TLR4 and calpain play pivotal roles in platelets function. Oxidative stress-mediated cytokines production was considered to be the main factor causing inflammation after cardiac surgery. The expression of many cytokines, including TNF-*α*, IL-6, and monocyte chemotactic protein- (MCP-) 1, increased during and after CPB [35]. Except for cytokine production, stimulations such as aortic-clamping ischemia, ischemia-reperfusion injury, blood cell attachment to the foreign surfaces of the oxygenator/CPB tube, and artificial surgical trauma may play important roles in postoperative inflammation; therefore, off-pump bypass graft operation significantly reduces oxidative stress and inflammation. Although clinicians still have differences of opinion on the advantages/disadvantages of the off-pump technique and conventional CPB for patients who undergo cardiac surgery [22, 23, 36, 37], at least, the patients in both the conventional CPB and off-pump groups had good early outcomes (little drainage loss, short ICU stay, and fever durations) in hospital as long as they expressed the active form of calpain after CABG surgery rather than the inactive form of calpain. This indeed indicates that maintaining active calpain is an important factor in patient recovery after cardiac surgery. In contrast, the patients with active calpain in the conventional CPB group still required larger blood transfusion volumes than the patients in the off-pump technique group, which may result from the cessation of pumping blood cells. Recently, we also undertook to distinguish the influences of foreign surface contamination by the CPB tube and oxygenator, surgical incisive trauma, and cardiac ischemia by comparing cytokine production and early outcome during conventional CPB technique and the off-pump technique in patients [26]. However, CPB-associated immune suppression results from the changes in TLR4 capacity on monocytes and neutrophils [38]. It is still remained to be elucidated that the causes result in TLR4 downregulation in platelets.

During the process of cardiac surgery with CPB, mechanical force (pressure from rolling pump) is the main cause resulting in platelet damage and hemolysis. Therefore, the transfusion of pRBC and fresh platelet is essential for the patients who are undergoing CPB. According to the results in Table 5, patients with CPB really receipted more transfusion of pRBC and platelet comparing to patients in off-pump technique group. Interestingly, patients with activated calpain in CPB group had similar level of early outcomes and platelet TLR4 expression to the patients in off-pump technique group. In contrast, even though the patients with inactivated calpain in CPB group receipted more platelet and pRBC transfusion, they also had worse early outcomes comparing to patients with activated calpain. Therefore, we cannot exclude the importance and effects of blood transfusion for patients with cardiac surgery in this study, and we still have the opinion that the calpain activation and TLR4 expression in platelets are associated with early outcomes.

Hyperglycemia and diabetes mellitus really induce oxidative stress which result in complex complications in many sickle situations [39]. The major finding of the previous study is that diabetes mellitus is associated with the activation of calpain [40]. Oxidative stress may upregulate calpain activity and induces apoptosis in pheochromocytoma cells [41]. Inhibition of calpain may reduce oxidative stress and attenuate endothelial dysfunction in diabetes [42]. However, the opposite evidence demonstrated that cardiac surgery induces oxidative stress, and oxidative stress may inhibit calpain activity [24]. In our results, mellitus patients (patient numbers 3, 5, and 10) also have inactive form of calpain after CPB. Although diabetes mellitus may be associated with patient's postsurgical outcomes, we also speculate that diabetes mellitus may not be a factor in regulation of platelets calpain activity in CPB.

Calpain is responsible for platelet shape change, clotting, and aggregation, which are mediated by the activation of cytoskeleton proteins [43]. Previous report demonstrated calpain with the important roles in regulating cytoskeletal signaling in vWf-activated platelets [44]. Calpain also regulates the activation of the extracellular fibrinogen-binding function of *α*IIb*β*3 and the platelet aggregation resulting in cleavage of integrin *β*3 [45]. Platelet-derived growth factor may suppress oxidative stress induced calpain activation in neurons [46]. Presently, the causes of inactive calpain in the majority of patients (82.35%) treated with conventional CPB are unknown; it is also unclear why 17.64% of the patients undergo calpain activation. There are many proteases with critical roles in the stability and assembly of cytoskeletal signaling complexes that may result in the phosphorylation of proteins, association of the receptor with the cytoskeleton, and a consequent increase in the calpain activity [47]. Whether the pressure force of pumping, aortic-clamping ischemia, ischemia-reperfusion injury, or attachment of the foreign CPB circuit during conventional CPB disrupts these normal conditions in platelets remains to be elucidated. Through further studies, we expect that patients' early outcomes may be improved and promoted by adjusting the activation of calpain in platelets after cardiac surgery with conventional CPB.

## 5. Conclusion

Our results highlight the important roles of TLR4 in blood coagulation and platelet function in clinic and in C57BL/6 mouse. Of clinical relevance, we also explored novel roles for calpain and TLR4 in platelets, which are associated with patients' early outcomes in cardiac surgery. These results also provide a basis for further controlling calpain activation and TLR4 expression as a therapeutic strategy to avoid coagulopathic and thrombocytic disorders after cardiac surgery.

## Figures and Tables

**Table 1:** Preoperative characteristics in CABG surgery patients.

	CABG surgery with conventional CPB	CABG surgery with off-pump technique
Total number	17	6
Age (years)	63.1 ± 10.1	61.5 ± 12.7
Body weight (kg)	69.3 ± 11.4	71.7 ± 14.5
Body height (cm)	162.3 ± 8.9	163.3 ± 9.2
Smoke (%)	23.5	50.0
Diabetes mellitus (%)	11.7	0
Hypertension (%)	64.7	66.7
Hypercholesterolemia (%)	82.4	83.3
COPD (%)	11.8	16.7
Stroke (%)	0	16.7
Peripheral vascular disease (%)	5.9	0
Prior myocardial infarction (%)	0	16.7
Ejection fraction (%)	59.7 ± 9.2	63.5 ± 8.3

COPD: chronic obstructive pulmonary disease. The values are shown as the mean ± SD.

**Table 2:** Perioperative characteristics in CABG surgery patients.

	CABG surgery with conventional CPB	CABG surgery with off-pump technique
Total number	17	6
CPB time (min)	119.1 ± 12.4	—
Aortic clamping time (min)	65.6 ± 5.2	—
Minimal esophageal temperature (°C)	26.1 ± 0.4	35.8 ± 0.3*
Heparin (units)	30476.2 ± 1708.0	28142.9 ± 4532.6
Number of grafts	3.1	2.5

CPB time indicates duration of CPB ischemia plus the duration of aortic cross clamping. The values are shown as the mean ± SD. *P < 0.05 compared with the group receiving CABG surgery with conventional CPB.

**Table 3:** Biochemical analyses in elective CABG patients.

Parameter	CABG surgery with conventional CPB (*n* = 17)		CABG surgery with off-pump technique (*n* = 6)	
	Presurgery	Postsurgery	Presurgery	Postsurgery
Blood (postsurgery: end of surgery)				
Neutrophil (%)	60.9 ± 9.2	85.9 ± 3.6*	64.7 ± 8.0	86.2 ± 4.3*
Eosinophil (%)	4.0 ± 2.6	2.1 ± 0.1	2.0 ± 1.4	2.2 ± 0.1
Basophil (%)	0.4 ± 0.3	0.5 ± 0.1	0.4 ± 0.2	0.5 ± 0.1
Monocyte (%)	6.4 ± 1.5	6.4 ± 1.9	6.7 ± 1.3	6.8 ± 1.6
Lymphocyte (%)	28.3 ± 9.2	7.5 ± 3.2*	26.1 ± 7.7	6.8 ± 2.7*
Platelet (×10^9^/L)	230.1 ± 91.1	111.2 ± 25.0*	188.8 ± 34.2	200.5 ± 22.5

Kidney function (postsurgery: after surgery for 24 h)				
BUN	17.4 ± 5.2	22.2 ± 8.1	17.5 ± 3.5	16.3 ± 4.5
Creatinine (mg/dL)	1.0 ± 0.4	3.0 ± 1.4	1.1 ± 0.3	1.3 ± 0.4

Liver function (postsurgery: after surgery for 24 h)				
AST	25.0 ± 10.1	39.7 ± 15.0	33.7 ± 13.2	42.0 ± 9.1
ALT	20.5 ± 13.5	22.6 ± 12.6	28.2 ± 13.7	33.2 ± 13.5

Myocardial damage (postsurgery: after surgery for 24 h)				
CK-MB/CK (%)	6.0 ± 3.2	4.7 ± 2.9	7.6 ± 5.5	4.1 ± 1.6
CK-MB (mg/dL)	22.9 ± 11.2	29.8 ± 13.9	22.7 ± 11.2	28.3 ± 7.5
Troponin I (mg/L)	1.0 ± 0.9	2.9 ± 2.0*	1.1 ± 1.6	1.6 ± 1.6

Inflammation (postsurgery: after surgery for 24 h)				
CRP (mg/L)	3.1 ± 2.7	5.5 ± 2.0	2.9 ± 2.8	5.6 ± 0.5

BUN: blood urea nitrogen; AST: glutamate oxaloacetate transaminase; ALT: glutamic pyruvic transaminase; CRP: C-reactive protein. The values are the mean ± SD. *P < 0.05 compared with presurgery at the same group.

**Table 4:** TLR4 expression, drainage, duration of ICU stay, and duration of fever in CABG surgery patients.

	CABG surgery with conventional CPB	CABG surgery with off-pump technique	*P* value
Total number	17	6	
TLR4 expression (% of preincision)	68.5 ± 5.6	89.0 ± 4.8	<0.05
Drainage (mL)	786.1 ± 84.8	534.7 ± 79.9	<0.001
pRBC transfusion (unit)	5.0 ± 0.5	1.1 ± 1.1	0.001
Platelet transfusion (unit)	1.3 ± 0.4	—	—
ICU stay (day)	4.4 ± 2.5	2.8 ± 2.0	0.171
ICU fever (hr)	23.5 ± 14.7	14.5 ± 7.6	0.173

ICU: intensive care unit. The values are shown as the mean ± SD. *P* < 0.05 was considered statistically significant.

**Table 5:** Calpain activity correlates with TLR4 expression and blood loss in postoperative CABG patients.

Calpain-1 expression after surgery	CABG surgery with conventional CPB		CABG surgery with off-pump technique		G1 versus G2 *P* value	G1 versus G3 *P* value	G2 versus G3 *P* value
	G1: active (patients 9, 12, 14, and 16) (*n* = 4, 23.53%)	G2: inactive (patients 1, 2, 3, 4, 5, 6, 7, 8, 10, 11, 13, 15, and 17) (*n* = 13, 76.47%)	G3: active (*n* = 6, 100%)	G4: inactive (*n* = 0, 0%)			
TLR4 expression (% of preincision)	83.92 ± 6.14	48.4 ± 19.3	89.0 ± 4.8	—	<0.05	0.157	<0.001
Drainage loss (mL)	528.0 ± 39.8	841.4 ± 96.7	534.7 ± 79.9	—	<0.05	0.897	<0.001
pRBC transfusion (unit)	3.5 ± 0.9	5.0 ± 1.0	1.1 ± 1.1	—	0.442	0.160	0.031
Platelet transfusion (unit)	0.9 ± 0.2	3.3 ± 2.3	0	—	0.001	0.008	<0.001
ICU stay (day)	2.3 ± 0.3	4.9 ± 0.7	2.8 ± 2.0	—	0.001	0.690	<0.001
ICU fever (hr)	16.3 ± 6.6	25.0 ± 4.1	14.5 ± 7.6	—	0.008	0.739	<0.05

TLR4: Toll-like receptor; pRBC: packed red blood cell; ICU: intensive care unit. The values are shown as the mean ± SD. *P* < 0.05 was considered statistically significant.

**Table 6:** ROTEM measurements of EXTEM in experimental mice.

Parameters	C57BL/6 (*n* = 6)	C57BL/6-*Tlr* ^*LPS* +/−^ (*n* = 6)	C57BL/6-*Tlr* ^*LPS* −/−^ (*n* = 6)
Coagulation activation and clot polymerization parameters			
Coagulation time (seconds)	31.00 ± 22.16	33.50 ± 18.27	30.67 ± 10.61
Clot formation time (seconds)	38.33 ± 9.61	44.00 ± 3.16	39.67 ± 7.63
*α*-angle (degree)	82.17 ± 2.04	78.17 ± 6.05	82.0 ± 1.55

Clot firmness parameters (mm)			
Amplitude at 5 min (A5)	59.33 ± 8.02	47.67 ± 7.15*	55.67 ± 5.28
A10	66.00 ± 6.63	56.17 ± 5.74*	62.67 ± 4.32
A15	69.00 ± 5.73	60.17 ± 5.23*	65.83 ± 4.17
A20	70.83 ± 5.34	62.17 ± 5.23*	67.67 ± 3.67
A25	71.83 ± 4.83	63.83 ± 5.00*	69.00 ± 3.52
A30	72.83 ± 4.83	64.83 ± 5.00*	69.67 ± 7.63
Maximum clot firmness (MCF)	74.67 ± 4.08	67.33 ± 4.97*	72.33 ± 2.16

ROTEM: rotation thromboelastometry; EXTEM: extrinsically activated assay using recombinant tissue factor.

The data are expressed as the median (interquartile range).*P < 0.05 compared with the C57BL/6 group.

**Table 7:** ROTEM measurements of FIBTEM in experimental mice.

Parameters	C57BL/6 (*n* = 6)	C57BL/6*- * *Tlr* ^*LPS* +/−^ (*n* = 6)	C57BL/6-*Tlr* ^*LPS* −/−^ (*n* = 6)
Coagulation activation and clot polymerization parameters			
Coagulation time (seconds)	19.83 ± 4.67	17.50 ± 6.89	12.83 ± 6.31*
*α*-angle (degree)	76.00 ± 7.56	78.50 ± 6.92	80.17 ± 2.32

Clot firmness parameters (mm)			
Amplitude at 5 min (A5)	14.33 ± 4.37	15.33 ± 4.59	17.76 ± 3.20
A10	15.33 ± 4.63	16.50 ± 5.58	19.33 ± 3.14
A15	16.50 ± 4.51	17.17 ± 5.53	20.67 ± 3.20*
A20	17.17 ± 4.36	17.67 ± 5.79	21.50 ± 3.33*
A25	17.50 ± 4.46	17.67 ± 5.79	21.83 ± 3.06*
A30	17.67 ± 6.11	17.83 ± 5.74	22.33 ± 3.20*
Maximum clot firmness (MCF)	18.33 ± 5.09	18.00 ± 6.26	23.33 ± 4.13*

ROTEM: rotation thromboelastometry; FIBTEM: extrinsically activated test using recombinant tissue factor with cytochalasin D. The data are expressed as the median (interquartile range). *P < 0.05 compared with the C57BL/6 group.

**Table 8:** The platelet component of clot strength in experimental mice.

	C57BL/6 (*n* = 6)	C57BL/6-*Tlr* ^*LPS* +/−^ (*n* = 6)	C57BL/6-*Tlr* ^*LPS* −/−^ (*n* = 6)
Maximum clot firmness (MCF)	58.80 ± 4.67	48.80 ± 3.27*	48.50 ± 3.00*
Maximum clot elasticity (MCE)	290.85 ± 10.99	190.89 ± 20.79*	218.37 ± 19.54*

The platelet component of clot strength was calculated using the following equation: MCF_platelet_ = MCF_EXTEM_  − MCF_FIBTEM_; MCE = (MCF × 100)/100 − MCF; MCE_platelet_  = MCE_EXTEM_  − MCE_FIBTEM_.*P < 0.05 compared with the C57BL/6 group.
